# 1300. Comparison of the Clinical Characteristics and Outcomes of Anaerobic Prosthetic Joint Infections (PJI) and Aerobic PJIs : A Retrospective Review

**DOI:** 10.1093/ofid/ofad500.1139

**Published:** 2023-11-27

**Authors:** Lemuel R Non, Poorani Sekar

**Affiliations:** University of Iowa Hospitals and Clinics, Iowa City, Iowa; University of Iowa Hospitals and Clinics, Iowa City, Iowa

## Abstract

**Background:**

PJI is an uncommon but serious complication of joint arthroplasties that can result in significant morbidity and cost. There is a paucity of studies in the literature on PJIs caused by anaerobic organisms either as the sole pathogen or part of a polymicrobial infection. The purpose of this study was to compare the clinical presentation and outcomes of PJI caused by anaerobes as opposed to aerobes.

**Methods:**

This was a retrospective review of 306 patients who met Musculoskeletal Infection Society (MSIS) criteria for PJI from 2014 to 2020 at the University of Iowa Hospitals and Clinics (UIHC). 38 patients with anaerobic PJI were compared to 268 patients with aerobic PJI. Statistical analyses were performed using Pearson’s Chi square, Fisher exact test, *t*-test, and binary logistic regression.

**Results:**

PJI with anaerobes represented 12.4% of all PJI cases in our institution, which is higher than the 3-6% reported in literature. Patients with anaerobic PJI had longer duration of symptoms (19.4 weeks vs 10.9 weeks, p = 0.012), were more likely to have a sinus tract on exam (23.7% vs 5.9%, p < 0.001), less fever on presentation ( 13.2% vs 30.2%, p = 0.029), more radiographic abnormalities (50.0% vs 31.1%, p< 0.001), lower erythrocyte sedimentation rate (49.0mm/hr vs 67.7 mm/hr, p = 0.003), lower C-reactive protein (6.6 mg/dL vs 12.1mg/dL, p = 0.004), and more likely to be polymicrobial (42.1%vs 8.0%, p < 0.001) compared to aerobic PJI. There was a higher proportion of anaerobes in shoulder PJIs compared to other joints (39.5% vs 5.5%, p< 0.001). Two-stage exchange was the most common surgical treatment in patients with anaerobic PJI (39.4%) whereas debridement and implant retention was the most common (44.5%) surgical treatment done in patients with aerobic PJI. No significant differences were seen between the two groups in terms of recurrences. Independent factors associated with anaerobic infection were shoulder prosthetic joint (AOR 10.5, CI 3.4 - 32.8) and presence of sinus tract (AOR 10.1, CI 3.1 - 32.6).

**Figure:**
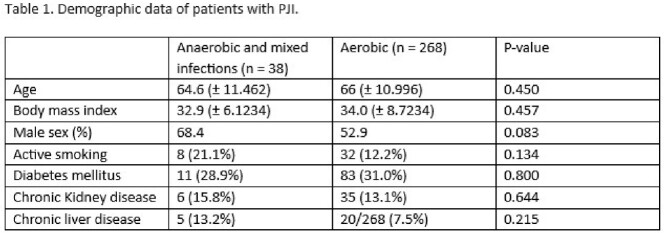

**Figure:**
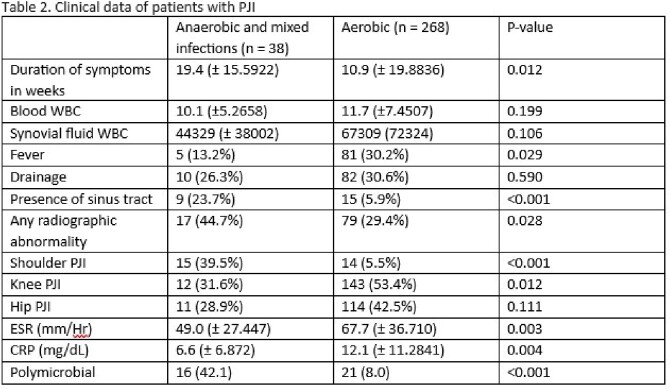

**Conclusion:**

Anaerobic cultures should be performed in patients with PJI, and they should especially be considered on the differential in patients with shoulder PJI, longstanding symptoms, sinus tract on examination and abnormalities noted on radiographs.

**Disclosures:**

**All Authors**: No reported disclosures

